# NMR structure of the protein NP_247299.1: comparison with the crystal structure

**DOI:** 10.1107/S1744309110005890

**Published:** 2010-07-06

**Authors:** Kristaps Jaudzems, Michael Geralt, Pedro Serrano, Biswaranjan Mohanty, Reto Horst, Bill Pedrini, Marc-André Elsliger, Ian A. Wilson, Kurt Wüthrich

**Affiliations:** a Department of Molecular Biology, The Scripps Research Institute, La Jolla, CA 92037, USA; b Joint Center for Structural Genomics, http://www.jcsg.org, USA; c Institute of Molecular Biology and Biophysics, ETH Zurich, CH-8093 Zurich, Switzerland; d Skaggs Institute of Chemical Biology, The Scripps Research Institute, La Jolla, CA 92037, USA

**Keywords:** structure comparison in crystals and in solution, structure-determination software, reference structures, nitrogenase iron–molybdenum cofactor

## Abstract

The NMR structure of the protein NP_247299.1 in solution at 313 K has been determined and is compared with the X-ray crystal structure, which was also solved in the Joint Center for Structural Genomics (JCSG) at 100 K and at 1.7 Å resolution. Both structures were obtained using the current largely automated crystallographic and solution NMR methods used by the JCSG. This paper assesses the accuracy and precision of the results from these recently established automated approaches, aiming for quantitative statements about the location of structure variations that may arise from either one of the methods used or from the different environments in solution and in the crystal. To evaluate the possible impact of the different software used for the crystallographic and the NMR structure determinations and analysis, the concept is introduced of reference structures, which are computed using the NMR software with input of upper-limit distance constraints derived from the molecular models representing the results of the two structure determinations. The use of this new approach is explored to quantify global differences that arise from the different methods of structure determination and analysis *versus* those that represent interesting local variations or dynamics. The near-identity of the protein core in the NMR and crystal structures thus provided a basis for the identification of complementary information from the two different methods. It was thus observed that locally increased crystallographic *B* values correlate with dynamic structural polymorphisms in solution, including that the solution state of the protein involves a slow dynamic equilibrium on a time scale of milliseconds or slower between two ensembles of rapidly interchanging conformers that contain, respectively, the *cis* or *trans* form of the C-terminal proline and represent about 25 and 75% of the total protein.

## Abstract 2

Comparison of the NMR and crystal structures of a protein determined using largely automated methods has enabled the interpretation of local differences in the highly similar structures. These differences are found in segments of higher *B* values in the crystal and correlate with dynamic processes on the NMR chemical shift timescale observed in solution.

## 1. Introduction

The hypothetical protein NP_247299.1 is the gene product of locus MJ0327 in the genomic sequence of the *Methanococcus jannaschii* genome (Bult *et al.*, 1996 ▶). Its function is unknown, but on the basis of sequence similarity the NP_247299.1 protein has been assigned to the dinitrogenase iron–molybdenum cofactor family (PF02579). This family includes NifB, NifX and NifY, all of which are involved in biosynthesis of the nitrogenase iron–molybdenum cofactor (FeMo-co) in nitrogen-fixing bacteria (Rubio *et al.*, 2002 ▶).

The crystal structure of NP_247299.1 (PDB code 2qtd) has pre­viously been solved by the Joint Center for Structural Genomics (JCSG). The NMR solution structure of NP_247299.1 has now been determined independently as part of a methods-development project in the JCSG. In this paper, we describe the NMR solution structure of NP_247299.1 obtained using a new protocol that was implemented by the JCSG NMR Core (see below) and present a systematic com­parison of the results obtained by largely automated solution structure determination with the crystal structure. In pursuing this comparison, we explore the use of reference crystal and NMR structures to investigate the influence of the different structure-determination software used by the two methods. The reference structures are computed from distance restraints derived from the crystal and solution molecular models, respectively, using the same simulated-annealing protocol as used for the computation and refinement of the experimental NMR structure. This study leads to a quantitative evaluation of the close global similarity of the protein core in the NMR and crystal structures and to detailed information on localized polymorphisms in the solution structure and their manifestation in the crystallographic data.

## 2. Materials and methods

### 2.1. Protein preparation

The plasmid vector pSpeedET containing the NP_247299.1 gene was obtained from the JCSG Crystallomics Core, where it had been generated using the polymerase incomplete primer extension (PIPE) cloning method (Klock *et al.*, 2008 ▶) to produce the protein for the crystal structure determination. For the NMR sample preparation, pSpeedET-NP_247299.1 was used as the template for PCR amplification with the primers 5′-CGGCAT*ATG* **GAAAACCTGTATTTT** **CAGGGA**ATAAATATGAAAGTAGCCATTTCAATG-3′ and 5′-CGGAAGCTT *TTA*TGGATTACTTATTTTACTTAATTCCCCCTCAATAAATAAAGAG-3′, where the *Nde*I and *Hin*dIII restriction sites are underlined and the initiation and stop codons are italicized. The forward primer contains an ENLYFQG TEV protease cleavage site (shown in bold). The PCR product was digested with *Nde*I and *Hin*dIII and inserted into the vector pET-28b between the same restriction sites after treatment with calf intestinal alkaline phosphatase (CIP). The resulting plasmid pET-28b-TEV-NP_247299.1 was used to transform *Escherichia coli* strain BL21 (DE3) (Novagen) and the protein was expressed in M9 minimal media containing either 1 g l^−1^ ^15^NH_4_Cl and 4 g l^−1^ unlabeled d-glucose or 1 g l^−1^ ^15^NH_4_Cl and 4 g l^−1^ [^13^C_6_]-d-glucose (Cambridge Isotope Laboratories) as the sole sources of nitrogen and carbon. After the addition of 100 mg l^−1^ kanamycin, the cells were grown at 310 K to an OD_600_ of 0.70, induced with 1 m*M* isopropyl β-d-1-thiogalactopyranoside (IPTG) and kept at 291 K for a further 20 h (final OD_600_ = 0.91). The cells were harvested at 5000*g* and 277 K for 10 min and freeze–thawed at 193 K for 15 min. The cell pellet was resuspended in 46 ml buffer *A* (20 m*M* sodium phosphate pH 7.5, 300 m*M* NaCl, 15 m*M* imidazole, 1 m*M* DTT) containing one Complete EDTA-free protease-inhibitor cocktail tablet (Roche) and lysed by ultrasonication. The soluble fraction of the cell lysate was isolated by centrifugation for 30 min at 20 000*g* and 277 K and passed through a 0.22 µm pore-size filter. The solution was then applied onto a 5 ml HisTrap HP column (GE Healthcare) pre-equilibrated in buffer *A*. The bound protein was eluted using a linear imidazole gradient from 15 to 500 m*M* over a 200 ml volume. Fractions containing the protein were pooled and treated with 25 µg ml^−1^ TEV protease for 17 h at 307 K in order to remove the 25-residue N-terminal expression and purification tag. The product was applied onto a HiPrep 26/10 desalting column pre-equilibrated in buffer *A*. The NP_247299.1 recovered from the flowthrough was reapplied onto a HisTrap HP 5 ml column pre-equilibrated in buffer *A*. Fractions containing the protein were concentrated to 10 ml by ultrafiltration using an Amicon ultracentrifugal filter device with 3 kDa molecular-weight cutoff (Millipore) and then applied onto a HiLoad 26/60 Sephacryl S-100 gel-filtration column (GE Healthcare) pre-equilibrated in NMR buffer (20 m*M* sodium phosphate pH 6.5, 1 m*M* DTT). The fractions containing NP_247299.1 were pooled and concentrated from 45 ml to 500 µl by ultrafiltration. All purification steps were monitored by SDS–PAGE. The yield of purified NP_247299.1 was 5.7 mg per litre of culture.

NMR samples were prepared by adding 5%(*v*/*v*) D_2_O and 0.03%(*w*/*v*) NaN_3_ to 500 µl of a 0.9 m*M* solution of ^15^N,^13^C-labeled NP_247299.1 in NMR buffer.

### 2.2. NMR spectroscopy

NMR experiments were conducted at 313 K on Bruker Avance 600 and 800 MHz spectrometers equipped with a TXI HCN *z*-gradient cryoprobe and an *xyz*-gradient room-temperature probe, respectively. 4D APSY-HACANH, 5D APSY-HACACONH and 5D APSY-CBCACONH data sets were recorded with 29, 32 and 24 projections, respectively (Hiller *et al.*, 2005 ▶, 2008 ▶). Three NOESY spectra were recorded with a mixing time of 60 ms: 3D [^1^H,^1^H]-NOESY-^15^N-HSQC, 3D [^1^H,^1^H]-NOESY-^13^C(ali)-HSQC and 3D [^1^H,^1^H]-NOESY-^13^C(aro)-HSQC. The ^13^C carrier frequencies were at 26 and 125 p.p.m. for the recording of the ^13^C(ali) and ^13^C(aro) data sets, respectively. A 3D HNHA experiment (Vuister & Bax, 1993 ▶) was recorded for the determination of ^3^ *J*_HNα_ coupling constants. Chemical shifts were referenced internally to the residual water signal. The chemical shift of the solvent water resonance relative to DSS was 4.61 p.p.m.

### 2.3. NMR structure determination

The polypeptide backbone resonance assignments were obtained from the aforementioned APSY-NMR experiments. The APSY-generated four- and five-dimensional peak lists were used as input for automated backbone assignment with the software *UNIO-MATCH* v.1.0.2 (Volk *et al.*, 2008 ▶). The backbone assignments were then interactively checked and completed. Side-chain resonance assignments were obtained with the program *UNIO-ATNOS*/*ASCAN* v.1.0.2 (Herrmann *et al.*, 2002*b* ▶; Fiorito *et al.*, 2008 ▶), using as input the aforementioned 3D ^15^N-resolved and ^13^C-resolved [^1^H,^1^H]-NOESY spectra. The assignments obtained from this automatic procedure were interactively checked and extended using the software *CARA* (Keller, 2004 ▶). NOE distance restraints were automatically collected using the same three NOESY data sets as for the side-chain assignment as input for the program *UNIO-ATNOS*/*CANDID* v.1.0.2 (Herrmann *et al.*, 2002*a* ▶,*b* ▶), which was used in combination with the NMR structure-calculation program *CYANA* v.3.0 (Güntert *et al.*, 1997 ▶). No explicit torsion-angle restraints were used in the input. A standard seven-cycle *UNIO-ATNOS*/*CANDID* protocol (Herrmann *et al.*, 2002*a* ▶) was employed, with 80 random starting conformers being subjected to a simulated-annealing schedule consisting of 8000 steps of torsion-angle molecular dynamics. The 40 conformers with the lowest residual *CYANA* target-function values after *UNIO-ATNOS*/*CANDID* cycle 7 were energy-minimized in a water shell with the program *OPALp* (Luginbühl *et al.*, 1996 ▶) using the AMBER force field (Cornell *et al.*, 1995 ▶). The 20 conformers with the lowest target-function values that satisfied the validation criteria (see below) were selected and analyzed using the program *MOLMOL* (Koradi *et al.*, 1996 ▶).

### 2.4. Structure validation and data deposition

Analysis of the stereochemical quality of the molecular models was accomplished using the PDB validation tools (http://www.pdb.org/), the JCSG Validation Central Suite (http://www.jcsg.org) and the *Verify*3*D* structure-validation server (http://nihserver.mbi.ucla.edu/Verify_3D/) in an in-house validation protocol used by the JCSG NMR Core (unpublished work). The chemical shifts have been deposited in BioMagResBank (accession No. 16389; http://www.bmrb.wisc.edu) and the atomic coordinates of the 20 conformers representing the NMR structure were deposited in the Protein Data Bank (accession code 2kla).

### 2.5. Calculation of reference crystal and reference NMR structures from proton–proton distance constraints derived from the crystal and NMR structures, respectively, using the same simulated-annealing protocol as used for the experimental NMR structure determination

In order to derive proton–proton distances from the X-ray crystal structure of NP_247299.1 (PDB code 2qtd), the positions of the H atoms were calculated using the standard residue geometry from the AMBER94 library in the software *MOLMOL* (Koradi *et al.*, 1996 ▶). All intra- and inter-residual distances shorter than 5.0 Å between pairs of H atoms were then extracted and those involving labile protons with fast chemical exchange (Wüthrich, 1986 ▶) were eliminated from the resulting list. The input of upper-limit distance bounds for the structure calculation was generated by increasing these proton–proton distances by 15%. This ‘loosening’ of the distance constraints is in line with the basic strategy of interpreting ^1^H–^1^H NOEs in terms of upper-limit distance bounds (Wüthrich, 1986 ▶); this procedure ensured good convergence of the simulated annealing and yielded identical structures as were obtained when using the actual distances as input. A bundle of 20 energy-minimized conformers representing the reference crystal structure was computed using the torsion-angle molecular-dynamics algorithm of the program *CYANA*, following the same selection protocol as for the experimental NMR structure determination.

To obtain the reference NMR structure, we followed a three-step protocol. (i) A list was prepared of all the ^1^H—^1^H distances shorter than 5.0 Å in the 20 conformers that represent the NMR structure. (ii) A new list was obtained that included the longest distance among the 20 conformers for each pair of H atoms in the list resulting from (i). (iii) The input of upper-limit distance bounds contained all the entries in list (ii) that were shorter than 5.75 Å [this value was empirically selected as the shortest cutoff that gave virtually identical results of the structure calculation to an input consisting of the complete list (ii)]. A bundle of 20 energy-minimized conformers was generated using the same selection criteria as for the reference crystal structure and the experimental NMR structure.

### 2.6. Comparison of the global displacements in the NMR structure, the reference NMR structure and the reference crystal structure with displacements calculated from the *B* values of the X-ray structure

Global displacements (Billeter *et al.*, 1989 ▶) of the backbone heavy atoms N, C^α^ and C′ in the bundles of 20 energy-refined conformers that were used to represent the NMR structure, the reference NMR structure and the reference crystal structure, *D*, were calculated using *MOLMOL* (Koradi *et al.*, 1996 ▶). For further interpretation, we used the average displacement per residue, 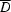, which is the arithmetic average of the *D* values for these three atoms per residue,
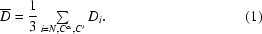
Similarly, we define the average crystallographic *B* value per residue, 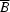, as 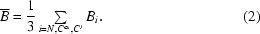
To formally express the precision of the crystal structure determination by 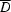 values, as used for the NMR structure and the two reference structures, we determined an empirical correlation coefficient, *c*, from a linear least-squares fit of the 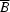 values in the experimental crystal structure to the corresponding 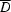 values in the reference crystal structure, so that the relation 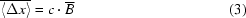
defines displacements per residue that correspond to the 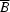 values. We thus do not address the question of comparing absolute values of 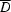 and 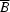 in the NMR structure and the crystal structure. In some ways (3) corresponds to the ‘inverse’ of previous approaches to derive ‘pseudo-*B* values’ from NMR displacements in attempts to obtain improved models for molecular replacement in crystal structure determinations (Weiss *et al.*, 1995 ▶; Wilmanns & Nilges, 1996 ▶).

### 2.7. Computation of global r.m.s.d. values, solvent accessibility and occluded surface packing (OSP)

Global r.m.s.d. values for the bundles of 20 conformers representing the NMR structure, the reference NMR structure and the reference crystal structure were computed with *MOLMOL* (Koradi *et al.*, 1996 ▶) using the mean coordinates as the reference. For each bundle of 20 conformers, r.m.s.d. values were computed for three atom selections, *i.e.* the backbone heavy atoms (bb), the core atoms with less than 15% solvent accessibility (co) and all heavy atoms (ha). For the crystal structure, the r.m.s.d. values for the three different atom selections were calculated according to 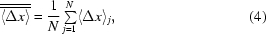
where *N* is the number of atoms selected for the superposition and 〈Δ*x*〉*_j_* was derived with (5) for each individual atom *j* from its crystallographic *B* value,
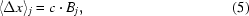
where *c* is the correlation coefficient determined for the backbone heavy atoms with (3) (see also Fig. 3*a*).

For comparison of different structures, each bundle was represented by the conformer with the smallest bb r.m.s.d. relative to the mean coordinates. R.m.s.d. values comparing different structures were then calculated for the atom selections bb, co and ha.

Solvent accessibility was only computed for the NMR structure, where mean values for the 20 conformers were obtained either for individual heavy atoms or for individual amino-acid residues. The rolling-sphere model implemented in the software *MOLMOL* was used, with a radius of 1.4 Å for the sphere representing the solvent molecule and a computation precision value of 3 (Koradi *et al.*, 1996 ▶).

The occluded surface packing (OSP) was computed with the *OS* software package (Pattabiraman *et al.*, 1995 ▶; http://www.csb.yale.edu/userguides/datamanip/os/), considering all heavy atoms in the polypeptide chain. Two different approaches were used. On the one hand, OSPs were evaluated for the crystal structure and for the conformers closest to the mean of the bundles representing the NMR structure and the two reference structures. In addition, OSPs were calculated for all 20 NMR conformers and the mean value and standard deviation were then evaluated.

## 3. Results

### 3.1. NMR structure of NP_247299.1

The automation of the determination of the NMR structure of NP_247299.1, as described in §[Sec sec2.3]2.3, provided the following results: the automated *UNIO-MATCH* routine yielded assignments for the ^1^H^N^, ^15^N, ^13^C^α^ and ^1^H^α^ atoms of 97 residues (94%) and for the ^13^C^β^ atoms of 91 residues. Interactive validation showed that the automated procedure had not generated any incorrect assignments and the backbone and ^13^C^β^ assignments were extended interactively to all 104 residues. On the basis of the complete backbone assignments, automated side-chain assignment with the program *UNIO-ATNOS*/*ASCAN* resulted in complete or partial assignments of the nonlabile H atoms of all 104 residues. Interactive inspection showed that about 95% of these assignments were correct and, for most of the side chains with partial assignment, the chemical shift lists could be expanded interactively. The time used for the interactive steps was about 70 h.

**Figure 1:**
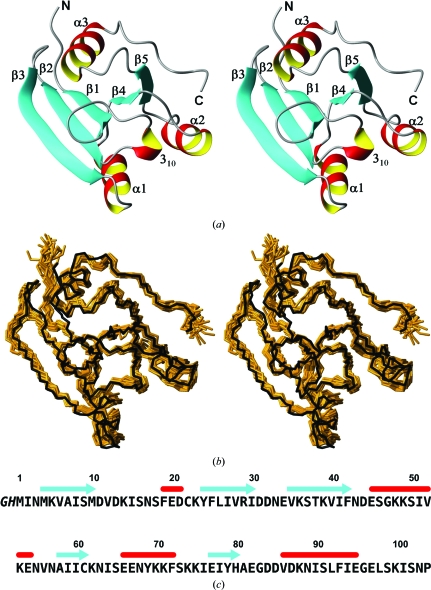
Amino-acid sequence and NMR structure of protein NP_247299.1 and comparison of the NMR structure with the crystal structure. (*a*) Stereo ribbon diagram of the NMR conformer closest to the mean coordinates of the bundle of conformers in (*b*). Color code: β-strands, cyan; helices, red/yellow; nonregular secondary structure, gray. The individual regular secondary structures are identified and the two chain ends are marked N and C. (*b*) Stereoview of a superposition of the polypeptide backbone heavy atoms of residues 1–102 of the crystal structure (black line) with the 20 conformers representing the NMR structure (brown). The crystal structure was superimposed for best fit with the mean atomic coordinates of the 20 NMR conformers. (*c*) Amino-acid sequence. Residues −2 and −1 originate from the expression and purification tag at the TEV cleavage site and are not part of NP_247299.1. The locations of regular secondary structures are indicated above the sequence.

The NMR structure of NP_247299.1 comprises three α-helices consisting of residues 45–54, 66–72 and 86–95, five β-strands formed by residues 4–10, 24–30, 35–42, 58–61 and 77–80 and a short 3_10_-helix for residues 19–21 in the sequential order β1–3_10_–β2–β3–α1–β4–α2–β5–α3 (Fig. 1 ▶ *a*). The longest polypeptide segment with nonregular secondary structure contains residues 11–18, but is well defined. The β-strands form a twisted sheet with topology 3–2–1–4–5, in which strands 3, 1, 4 and 5 are parallel to each other and strand 2 is antiparallel (Fig. 1 ▶ *a*). Helices α1 and α2 are arranged in parallel at a small angle, with the 3_10_-helix positioned between them at their N-­terminal ends. This combination of three helices is docked against one side of the twisted β-sheet, whereas helix α3 is located on the opposite side. Statistics of the NMR structure determination are given in Table 1 ▶.

The fold topology of NP_247299.1 resembles a ribonuclease H-like motif, with a three-layer α/β/α architecture. A *DALI* search (Holm *et al.*, 2008 ▶) revealed eight structures with a *Z* score of ≥10, which indicates strong fold similarity. Seven of these proteins belong to the COG1433 protein family, which contains 28 functionally uncharacterized conserved proteins from 13 different genomes, including NP_247299.1. Superposition of the previously reported structures of six COG1433-family proteins [PDB codes 2re2 (Joint Center for Structural Genomics, unpublished work), 1rdu (Etezady-Esfarjani *et al.*, 2004 ▶), 1o13 (Joint Center for Structural Genomics, unpublished work), 1t3v (Columbus *et al.*, 2005 ▶), 2yx6 (T. Hosaka, K. Murayama, T. Terada, M. Shirouzu & S. Yokoyama, unpublished work) and 1eo1 (Cort *et al.*, 2000 ▶)] with NP_247299.1 confirmed high conservation of the relative spatial arrangement of the regular secondary structures, with any structure variations being limited to the connecting nonregular polypeptide segments, where they appear to correlate with amino-acid insertions and deletions.

### 3.2. Strategy for structure comparisons

During the last decade, the JCSG has developed novel largely automated protocols for NMR structure determination (unpublished work) and crystal structure determination, the latter of which has been found by others to yield the highest quality crystal structures presently deposited in the PDB (Brown & Ramaswamy, 2007 ▶). Here, we compare the accuracy and precision of NMR and X-ray structures of the protein NP_247299.1 that were independently determined with these new approaches. Since the two structures were found to have a virtually identical protein core, this study can furnish a detailed evaluation of local structure variations that may be associated with the different environments in solution and in single crystals. We established a frame of reference for this comparison that would monitor the possible impact of the different software used for the structure calculation and refinement by the two techniques. To this end, we used the NMR software to compute a reference crystal structure and a reference NMR structure based on an input of upper distance constraints derived from the corresponding experimental structures, as described in §[Sec sec2]2. This approach was validated by checking that all of the experimentally observed NOE cross-peaks coincide with peak positions contained in the structure-derived input. We then explore the use of these two reference structures to support the evaluation of the significance of the few apparent differences between the experimental NMR and crystal structures. While systematic comparisons of crystal structures and NMR structures have been carried out for many years (see, for example, Billeter *et al.*, 1989 ▶; Braun *et al.*, 1989 ▶, 1992 ▶; Hyberts *et al.*, 1992 ▶; Yang *et al.*, 2007 ▶), owing to the advancement of the two methodologies and the automated methods that can reduce human error or bias, we can now focus on more subtle differences between the NMR and crystal structures that might in some instances also relate to the biological function. The identification of such locally variable sites is guided by a search for sequence locations with high *B* values in the crystal structure or/and high variation within the bundle of 20 NMR conformers. Characterization of these local ‘hot spots’ is then supported by additional NMR measurements.

### 3.3. Global fold comparisons

The NMR structure of NP_247299.1 was solved at 313 K using 0.9 m*M* protein solution in 20 m*M* sodium phosphate buffer pH 6.5 containing 1 m*M* DTT and 0.03%(*w*/*v*) NaN_3_. The crystal structure was determined to 1.7 Å resolution at 100 K using a crystal obtained at 277 K from 100 m*M* Tris–HCl solution pH 7.0 containing 50%(*w*/*v*) PEG 200. Here and in §[Sec sec3.4]3.4 we identify differences between the results of these two structure determinations.

The reference structures were calculated from a significantly larger number of upper-limit distance constraints than the experimental NMR structure. The main factors causing the numbers of constraints to differ (Table 1 ▶) are that, owing to the limited resolution and sensitivity of the NMR experiments, only a fraction of the short ^1^H—^1^H distances are collected in the solution structure determination, whereas in the aforementioned molecular models all the short contacts are evaluated. Furthermore, in the present reference structure calculations only the methyl groups were represented by pseudo-atoms (Wüthrich *et al.*, 1983 ▶), whereas in the experimentally collected input the methylene groups and the pairs of symmetry-related ring protons of Phe and Tyr were also represented by pseudo-atoms.

**Figure 2:**
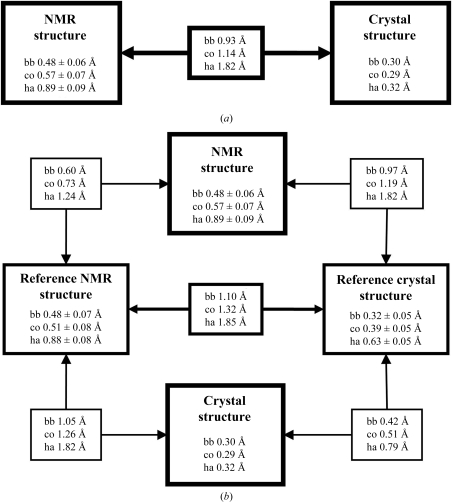
Analysis of the crystal, NMR and reference crystal and NMR structures. (*a*) R.m.s.d. values describing the precision of structure determinations of NP_247299.1 by NMR in solution at 313 K or by X-ray diffraction in crystals at 100 K and pairwise comparisons of the two experimentally determined structures. The atoms used for the comparisons are bb, backbone atoms N, C^α^ and C′; co, core heavy atoms defined as having less than 15% solvent accessibility; ha, all heavy atoms. These three atom-type selections were superimposed for best fit of residues 1–102 to compute the r.m.s.d. values. (*b*) Corresponding data as in (*a*) for the reference NMR structure and the reference crystal structure and for pairwise comparisons with the experimental structures. In (*a*) and (*b*), numbers framed by thick lines represent the precision of the experimental NMR and crystal structures and their comparison. For the crystal structure, ‘global deviations’ corresponding to the r.m.s.d.s were computed from the experimental *B* values using (2)–(5) [Disp-formula fd3] [Disp-formula fd4] [Disp-formula fd5]. For the structure comparisons, r.m.s.d. values were computed between the crystal structure coordinates and those of the conformer closest to the mean atomic coordinates of each of the three ensembles of 20 conformers that represent the NMR structure and the two reference structures. Numbers framed by medium lines represent the precision of the reference NMR and reference crystal structures and their comparison and thin frames contain the comparisons between experimental and reference structures.

The high global structure similarity between the NMR and X-ray structures of NP_247299.1 is visualized by superposition of the crystal structure and the bundle of NMR conformers (Fig. 1 ▶ *b*). A quanti­tative comparison yielded backbone (bb) and all-heavy-atom (ha) r.m.s.d. values of 0.93 and 1.82 Å, respectively, between the NMR conformer closest to the mean coordinates of the bundle of 20 conformers and the crystal structure (Fig. 2 ▶). To assess the significance of these r.m.s.d. values between the experimental data, we use the aforementioned reference structures as a frame of reference. We conclude from the following observations that the two reference structures provide a valid basis for this work: comparison of the two reference structures yields similar r.m.s.d. values to those between the experimental structures and the closest similarities prevail on the one hand between the crystal structure and the reference crystal structure and on the other hand between the NMR structure and the reference NMR structure. The crystal structure and the reference crystal structure exhibit nearly identical r.m.s.d.s relative to the experimental NMR structure.

Fig. 2 ▶ also includes information on the precision with which the experimental structures and the reference structures are defined. Not surprisingly, although our approach does not warrant a quantitative comparison of the r.m.s.d. values for the two experimental structures, it appears that overall the crystal structure determined at 100 K is more precisely defined than the 313 K NMR solution structure. The following observation on the treatment of the crystal structure with the NMR software is of special interest: although the average displacement calculated with (4) for all heavy atoms in the crystal structure, 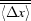 = 0.32 Å, is essentially identical to the value obtained for the backbone heavy atoms, 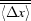 = 0.30 Å, the r.m.s.d. values for the corresponding selections of atoms in the reference crystal structure differ by nearly twofold, which is similar to the corresponding relations in the NMR structure and the reference NMR structure.

Overall, the preliminary conclusions from the data collected in Fig. 2 ▶ are that the global structural properties of the polypeptide backbone and the core atoms with solvent accessibility below 15% are nearly identical in the crystal and solution structures and that the larger r.m.s.d. values calculated for the all-heavy-atom comparisons can be almost entirely attributed to the solvent-accessible segments of the polypeptide chain. The data of Fig. 2 ▶ will be further analyzed in §[Sec sec4]4 based on comparison of the structural details in the four molecular species represented in the figure.

### 3.4. Comparison of structural details

In this section, we extend the global structure comparisons of Fig. 2 ▶ by evaluation of selected per-residue parameters, which we then consider along the amino-acid sequence. All of these comparative studies use the crystal structure atomic coordinates and/or the bundles of 20 conformers that represent the NMR structure and the two reference structures (Fig. 2 ▶). In instances where the bundles of 20 conformers are represented by a single conformer, the conformer with the smallest global backbone r.m.s.d. value relative to the mean coordinates of the bundle is used.

#### 3.4.1. Precision along the amino-acid sequence

**Figure 3:**
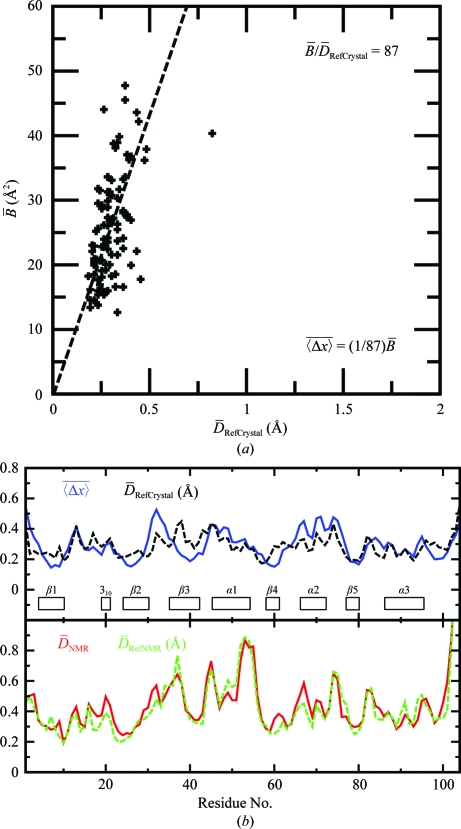
Comparison of crystallographic *B* values and NMR displacements (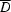). (*a*) Linear least-squares fit of the crystallographic 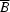 values (2) *versus* the corresponding displacements 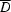 (1) in the reference crystal structure, yielding the *c* value in (3) for NP_247299.1, with *c* = 1/87. (*b*) Plots of the per-residue polypeptide backbone displacements *versus* the NP_247299.1 sequence. Upper panel, crystal structure and reference crystal structure. Lower panel, NMR structure and reference NMR structure. For the crystal structure, per-residue displacements were calculated from the 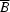 values with (2) and (3). For the NMR structure and the two reference structures, the data correspond to the per-residue displacements calculated with (1). The locations of regular secondary structures are indicated at the bottom of the upper panel.

To represent the precision of the NMR structure and the two reference structures, we use the per-residue displacement, 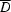, as defined by (1). For the crystal structure, the per-residue displacement 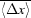 is used as calculated from the 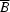 values with (2) and (3). Fig. 3 ▶(*a*) illustrates the empirical determination of the coefficient *c* in (3) by a linear fit of the crystallographic 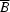 values to the 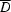 values of the reference crystal structure. Fig. 3 ▶(*b*) shows plots of the displacements *versus* amino-acid sequence for the NMR structure, the crystal structure and the two reference structures analyzed in Fig. 2 ▶. For the NMR structure, the profile of the plot of displacements *versus* the sequence is very closely mimicked by the reference NMR structure. Somewhat larger variations are observed between the crystal structure and the reference crystal structure, which probably reflects the use of different software for the refinement of these two molecular species. For a large part of the sequence, there is also a close coincidence of the qualitative features of these profiles between the experimental NMR and crystal structures. For example, there is a good correlation of small displace­ments with the positions of the β-strands. For the α-helical regions, the displacements vary between lower and higher values, with an indication of three- to four-residue repeats. Since the helices are flanked by the β-sheet on one side and exposed to the solvent on the other (Fig. 1 ▶ *a*), these periodic variations of the displacements appear to correlate with the side-chain solvent accessibility. The close similarity of the displacement profiles for the experimental structures and the reference structures is remarkable, confirming that the NMR software reproduces the experimental structures in the reference crystal structure and the reference NMR structure (see also §[Sec sec2.5]2.5). Relatively high displacements are observed for residues 10–15, 30–34, 44–48 and 66–75 in the crystal structure and for residues 35–39, 42–46, 52–56 and 73–75 in the NMR structure.

Overall, the data in Fig. 3 ▶(*b*) show that the small global r.m.s.d. values for the pairwise comparisons of the two experimental structures and the two reference structures in Fig. 2 ▶ are paralleled by close coincidence of the per-residue displacements along nearly the entire sequence. The aforementioned short polypeptide segments with above-average displacement values either in the NMR or the crystal structure will be used as a lead for investigating possible local differences between the protein structures in solution and in the crystal (see §[Sec sec4]4).

#### 3.4.2. Backbone dihedral angles

**Figure 4:**
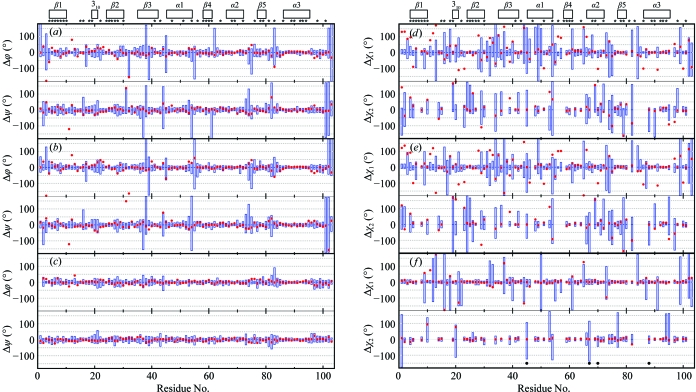
Variation in backbone dihedral and side-chain torsion angles and comparisons with the crystal structure. (*a*–*c*) Spread of the values for the backbone dihedral angles ϕ and ψ in the bundles of 20 conformers representing the NMR structure (*a*), the reference NMR structure (*b*) and the reference crystal structure (*c*) of NP_247299.1 (Fig. 2 ▶) and comparisons with the crystal structure. In this presentation, the mean value in the bundles of 20 conformers is at 0°, the blue vertical bars represent the spread of the values within the bundles and the red dots indicate the deviation of the crystal structure values from the corresponding mean values for the bundle of 20 conformers. (*d*–*f*) Spread of the values for the amino-acid side-chain torsion angles χ_1_ and χ_2_ in the NMR structure (*d*), the reference NMR structure (*e*) and the reference crystal structure (*f*) of NP_247299.1 (Fig. 2 ▶) and comparison with the crystal structure. At the top, the locations of the regular secondary structures are indicated and asterisks identify the residues with solvent accessibility below 15% in the NMR structure. Filled circles at the bottom of (*f*) indicate four residues for which the side chains were truncated in the crystal structure because they were not observed in the electron-density maps.

Overall, most of the dihedral angles in the NMR structure are defined with high precision and coincide closely with the X-ray structure, as shown in Figs. 4 ▶(*a*), 4 ▶(*b*) and 4 ▶(*c*). In this figure, we use a presentation of the protein dihedral angles that was adapted from Hyberts *et al.* (1992 ▶). The ranges of ϕ and ψ about the mean values in the ensembles of 20 conformers are represented by blue bars. Red dots represent deviations of the dihedral angle values in the crystal structure from the corresponding mean values of the bundles of 20 conformers. Fig. 4 ▶(*a*) presents the ϕ and ψ data for the experimental NMR structure. Excluding the two chain-terminal pentapeptide segments, only nine residues have a spread exceeding ±60° of the ϕ-angle values among the 20 NMR conformers. All of these, except for Thr38 and Lys39, which will be discussed in §[Sec sec4.4]4.4, are located in loops or turns and Gly83 is the only one with solvent accessibility below 15%. The sizeable spread of its ϕ angle occurs in concert with a large ψ spread for Glu82. Comparison with the crystal structure shows that nearly all ϕ and ψ dihedral angles in the crystal fall within the range covered by the 20 NMR conformers. The only large differences are seen for the ϕ values of Asn3 and Val12 and the ψ value of Asp11. In addition, deviations of more than 15° from the range covered by the NMR conformers are found for ϕ of Lys5, Lys23, Lys75 and Glu97 and for ψ of Gly47. Four other residues have ϕ and ψ values in the crystal structure that are within 15° of the ranges covered by the 20 NMR conformers but for which at least one of the dihedral angles deviates by more than 60° from the mean of the NMR conformers, *i.e.* Asp31, Asp32, Glu82 and Gly83.

Comparison of the reference NMR structure with the crystal structure (Fig. 4 ▶ *b*) reproduces the data seen in the comparison of the NMR and crystal structures in Fig. 4 ▶(*a*). In contrast, the reference crystal structure shows a large spread of the backbone dihedral angles only for Glu82 and Gly83 (see above; Fig. 4 ▶ *c*). For all other residues, the dihedral angles are precisely defined by the crystallographic data when re-evaluated with the use of the NMR software. In conclusion, we observe that the high accuracy of the backbone conformation in the crystal structure is matched by about 90% of the polypeptide chain in the solution structure. In §[Sec sec4]4, the few outliers will be used as a lead for analyzing possible local differences between the NMR and crystal structures.

#### 3.4.3. Side-chain torsion angles

Data on the side-chain torsion angles (Figs. 4 ▶ *d*, 4 ▶ *e* and 4 ▶ *f*) are presented in a format corresponding to the presentation of the backbone dihedral angles. A remarkably high coincidence is again found between the NMR and crystal structures, with 34 of 40 hydrophobic core side chains (asterisks in Figs. 4 ▶ *d*, 4 ▶ *e* and 4 ▶ *f*) having the same χ_1_ values in the crystal and NMR structures. However, for about 20% of the residues, there are significant differences in the side-chain torsion angles between the crystal structure and the mean values of the bundle of NMR conformers. For the solvent-accessible residues, the side-chain torsion angles in the bundles of 20 conformers representing the NMR structure and the two reference structures show quite large spreads. It is also apparent that comparisons of the NMR structure and the reference NMR with the crystal structure yield similar results. As will be explained in §[Sec sec4]4, it is of interest for interpretation of the data in Fig. 4 ▶(*d*) that the reference crystal structure shows large spreads for the χ_1_ angles of Ser, Asp and Cys residues and large χ_2_ values for Glu.

#### 3.4.4. Occluded surface packing

**Figure 5:**
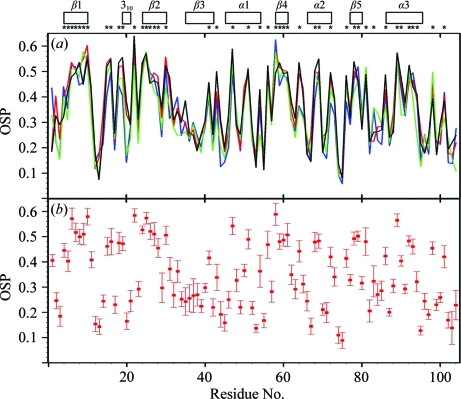
Surface packing along the polypeptide chain. (*a*) Plots *versus* the amino-acid sequence of the per-residue occluded surface packing (OSP, a dimensionless quantity covering the range 0.0–1.0; Pattabiraman *et al.*, 1995 ▶) for the NMR structure, the crystal structure and the two reference structures of NP_247299.1 (Fig. 2 ▶). For the NMR structure and the two reference structures, the OSP value for the conformer closest to the mean atomic coordinates are shown. Color code: NMR structure, red; crystal structure, blue; reference NMR structure, green; reference crystal structure, black. At the top, the locations of the regular secondary structures are indicated and asterisks identify the residues with solvent accessibility below 15% in the NMR structure. (*b*) Plot *versus* the amino-acid sequence of the mean per-residue OSP values in the NMR structure and the standard deviations among the 20 conformers (red).

Plots of the occluded surface packing (OSP; Pattabiraman *et al.*, 1995 ▶) per residue *versus* the sequence are displayed in Fig. 5 ▶(*a*), where the NMR structure and the two reference structures are represented by the conformer closest to the mean coordinates of the structure bundles. In addition, Fig. 5 ▶(*b*) reports the mean per-residue OSP values and the standard deviations for the bundle of 20 conformers, which shows that the spread of the OSP values for the individual residues is small when compared with the variations along the sequence. Notwithstanding small quantitative variations, the NMR and crystal structures, as well as the two reference structures (Fig. 2 ▶), all display the same OSP profiles, including low packing of the central polypeptide segment of residues 32–41, reduced packing near the two chain ends and near-identical extreme values for individual residues. On a general note, it is apparent that high OSP values correlate with low solvent accessibility and, within limits, also with the assignment of regular secondary structures.

## 4. Discussion

**Figure 6:**
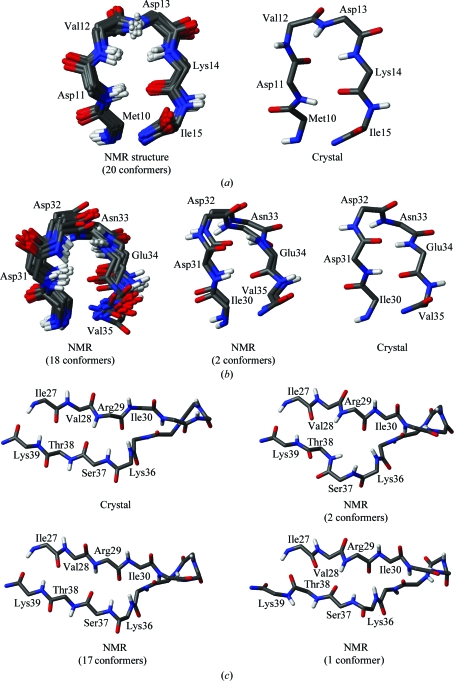
Stick representations of polypeptide segments in the NMR structure and in the crystal structure that show local variation as discussed in the text. C, N, O and H atoms are colored black, blue, red and gray, respectively. (*a*) Residues 10–15. (*b*) Residues 30–35. (*c*) Residues 27–39, which undergo a dynamic process involving residues 36–39, as evidenced by the NMR data in Fig. 7 ▶.

The key message is that the two structures of NP_247299.1 determined with current JCSG methodology either in solution at 313 K for NMR data collection or in a single crystal at 100 K show very close coincidence both globally (Fig. 2 ▶) and in residue-by-residue com­parisons (Figs. 3 ▶, 4 ▶ and 5 ▶). The extensive overall similarity of the two structures now provides a basis for investigations of subtle local structure variability. This approach is supported by the reference NMR structure, the reference crystal structure (Fig. 2 ▶) and supplementary NMR measurements, in addition to those of our standard structure-determination protocol (see Figs. 6 ▶, 7 ▶ and 8 ▶ below).

### 4.1. Global comparisons

We introduced the concept of reference structures and explored its use in support of the evaluation and comparison of the experimental NMR and crystal structures. The precision of the reference NMR structure is essentially identical to that of the experimental NMR structure (Fig. 2 ▶), confirming that the limited data set that can be collected in a structure-quality protein solution contains sufficient information to achieve nearly identical precision of the structure determination as would be obtained from the complete set of distance constraints. As we have pointed out previously, the precision of the reference crystal structure differs by about twofold when considering either the backbone heavy atoms or all heavy atoms, whereas the experimental crystal structure shows nearly identical global r.m.s.d. values for these two selections of atoms (Fig. 2 ▶). We rationalize this apparent difference by the facts that on the one hand the experimental crystal structure is subject to intermolecular contacts in the crystal lattice and side-chain atoms that were poorly defined in the electron-density maps were not included and their side chains were truncated (identified in Figs. 4 ▶ *d*, 4 ▶ *e* and 4 ▶ *f*). On the other hand, calculation of the reference crystal structure using the NMR software *CYANA* v.3.0 (Güntert *et al.*, 1997 ▶) and *OPALp* (Luginbühl *et al.*, 1996 ▶) is performed with a single molecule embedded in a water bath. We further rationalize the approximately 30% higher precision of the reference crystal structure when compared with the reference NMR structure by the fact that the X-ray data were collected at a much lower temperature than the NMR data.

Overall, we conclude from these considerations that the combination of crystal structure and reference crystal structure provides a robust platform for comparative studies with the solution NMR structure. In particular, the availability of the reference crystal structure helps to distinguish between effects from the different protein environments in the crystal and in solution and from possible bias arising from the use of different software for the refinement of the two experimental structures. A general conclusion from the global comparisons in Fig. 2 ▶ and the data on individual amino-acid residues in Figs. 3 ▶, 4 ▶ and 5 ▶ is that the polypeptide segments of NP_247299.1 with solvent accessibility below 15% can be near-identically superimposed in the NMR and crystal structures, while larger structure variations are indicated for some of the more highly solvent-exposed polypeptide segments.

### 4.2. Implications of high crystallographic *B* values in discrete polypeptide segments

Along the polypeptide chain, high *B*-value-derived displacements (3) are noted for four segments (residues 10–15, 30–34, 44–48 and 66–74). Segments 10–15 and 30–34 in the NMR structure are further explored here. The high *B* values in the other two segments relate to independently observed dynamic features of the NMR structure, as described in §4.4 and §4.6.

In the NOE-based NMR structure, residues 10–15 form a tight turn, with the peptide bond Asp11–Val12 flipped by 180° when compared with the crystal structure (Fig. 6 ▶ *a*). This local feature in the NOE-­based NMR structure is sterically unfavorable. We, therefore, collected additional NMR data that would be differently averaged in a dynamic conformational ensemble than the ^1^H–^1^H NOEs, *i.e.* scalar amide proton–α-proton spin–spin couplings, ^3^ *J*_HNα_ (Table 2 ▶). For Val12, a ^3^ *J*_HNα_ value of 7.1 Hz was measured. Using the Karplus relation for this coupling constant (Wüthrich, 1986 ▶), we estimate that the corresponding values in the crystal and NMR structures of Fig. 6 ▶(*a*) would be 8.9 and 4.5 Hz, respectively. We conclude that the experimental value of 7.1 Hz corresponds to a weighted average owing to conformational exchange between two or multiple locally different conformers present in solution. The *r* ^−6^-weighted average of the NOE distance constraints corresponding to these rapidly interchanging conformers thus resulted in a spurious sterically unfavorable local structure which is not compatible with the additional ^3^ *J*_HNα_ data. In this instance, the high *B* values of this region in the crystal structure led us to discover a local dynamic feature in the NMR structure obtained using our standard protocol, in which conformational averaging was taking place on the sub-millisecond time scale.

Residues 30–34 again form a tight turn in the crystal structure which is also seen in two of the 20 NMR conformers. A second form, which is present in 18 of the 20 NMR conformers, has the Asp31–Asp32 peptide bond rotated by 180° (Fig. 6 ▶ *b*). Similar to the situation represented in Fig. 6 ▶(*a*), this leads to a sterically unfavorable local conformation. Although, in this case, the ^3^ *J*_HNα_ values do not provide equally clear evidence, we conclude that the high *B* values in the crystal structure again correlate with a spurious local structural detail that results from dynamic averaging of ^1^H–^1^H NOE distance con­straints.

### 4.3. Implications of high displacements in a polypeptide segment of the NMR structure

The segment corresponding to residues 52–56 has the highest displacements in the NMR structure, with particularly low precision for Glu54 and Asn55. We attribute this locally low precision to limited experimental NOE data for three adjacent hydrophilic residues on the solvent-exposed face of helix α1. Segment 53–55 has on average only 14 NOE constraints per residue, compared with the mean value for the entire polypeptide chain of 24 constraints per residue.

### 4.4. Implications of variable backbone dihedral angles

**Figure 7:**
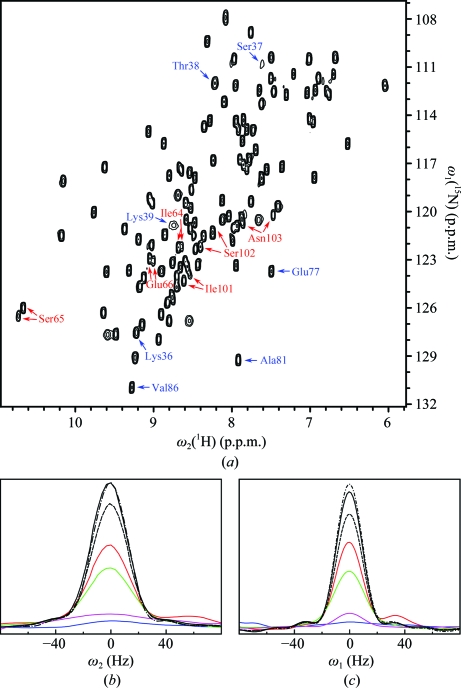
NMR evidence for slow conformational exchange between several locally different conformations formed by the polypeptide segment 36–39 (see also Fig. 6 ▶ *c*) and for the coexistence of two conformational species with distinct NMR signals for the polypeptide segments 64–66 and 101–104 (see also Fig. 8 ▶). (*a*) 2D [^15^N,^1^H]-HSQC spectrum of a 0.9 m*M* solution of NP_247299.1 recorded at 800 MHz and 313 K. The cross-peaks of the residues involved in the aforementioned conformational polymorphisms are identified with the following color code: blue, residues 36–39 and, for reference, Glu77, Ala81 and Val86 [see (*b*) and (*c*) below]; red, residues 64–66 and 101–103, which all show two signals (see Fig. 8 ▶). (*b*, *c*) NMR line-shape analysis reveals slow conformational exchange between the different conformations of the polypeptide segment 36–39 shown in Fig. 6 ▶(*c*). (*b*) and (*c*) show cross-sections along ω_2_(^1^H) and ω_1_(^15^N), respectively, illustrating pronounced line broadening of the cross-peaks belonging to Lys36 (red), Ser37 (blue), Thr38 (green) and Lys39 (magenta) when compared with the reference peaks of Glu77 (black), Ala81 (black dashed line) and Val86 (black dashed/dotted line).

The segment consisting of residues 36–39 attracted attention since it shows the only large variations of backbone dihedral angle values within regular α or β secondary structure. We noticed that the position of a β-bulge in strand β3 varies among the 20 NMR conformers (Fig. 6 ▶ *c*) and involves rearrangement of the hydrogen-bonding network. In the crystal structure and in 17 of the 20 conformers, a classic β-bulge is observed spanning residues 36–37. Two NMR conformers show a conformation with residue 38 bulged out. One NMR conformer shows a conformation with the peptide bond between Thr38 and Lys39 flipped by 180° and Lys39 and Val40 bulged out. It is quite intriguing that the reduced occluded surface-packing values observed in this region for both the NMR and the crystal structures (Fig. 5 ▶ *a*) would appear to allow local rearrangements. A qualitative line-shape analysis of the H^N^ resonances of residues Ser36–Lys39 confirms conformational fluctuations on the millisecond timescale, which is evidenced in severe line broadening of peaks in the 2D [^15^N,^1^H]-HSQC spectrum (Fig. 7 ▶). Additional NMR measurements at 298 K showed more pronounced line broadening for the resonances in Figs. 7 ▶(*b*) and 7 ▶(*c*), confirming that we observe exchange broadening of the averaged signals of the exchanging conformers (Wüthrich, 1986 ▶).

### 4.5. Side-chain dihedral angles and packing density

The comparison of side-chain dihedral angles (Figs. 4 ▶ *d*, 4 ▶ *e* and 4 ▶ *f*) showed that large spreads of χ_1_ and χ_2_ values in the NMR structure occur more frequently than for the backbone angles, which could be attributed in the first instance to the higher degree of freedom of peripheral side chains in solution. An initial clue to the interpretation of the lower precision of side-chain torsion angles also comes from the fact that the reference crystal structure shows large spreads for the χ_1_ angles of several Ser, Asp and Cys residues and for the χ_2_ angles of Glu residues, which do not include non-labile H atoms beyond the β- or γ-methylene positions, respectively, and are therefore not constrained during the NMR structure-calculation protocol. As the conformations of the side chains determine how the protein is packed, we looked for a correlation between the spread of χ angles and the variation in packing density. However, as shown in Fig. 5 ▶(*b*), the standard deviations for the occluded surface-packing values in the NMR structure have similar small values for all residues, indicating that the lower precision of χ angles in the NMR structure has no substantial impact on packing density. As large changes in side-chain conformations would particularly affect the packing of core residues, we analyzed the definition of the χ_1_ and χ_2_ angles of Ile, Leu and Val residues, which are usually located in the protein core. For 22 of 26 Ile, Leu and Val residues, the χ_1_-angle spread is less than 60°, where all of the outliers are Val. For 12 of 18 Ile and Leu residues, the χ_2_-angle spread is less than 60°. This indicates a certain level of plasticity in the core of the protein that allows local rearrangements of internal side chains, such as the flipping of particular isopropyl groups, without affecting the packing density. For solvent-exposed peripheral side chains, the packing is low in general and is not further affected by large spreads of χ_1_ and χ_2_ angles.

### 4.6. C-terminal Asn–Pro *cis*–*trans* isomerization in the NMR structure

**Figure 8:**
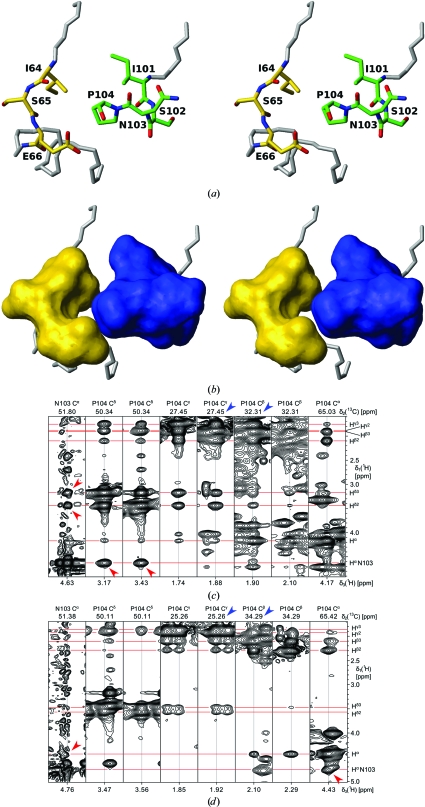
*Cis*–*trans* isomerization of Pro104. (*a*) Stick representation of all heavy atoms of the polypeptide segments 64–66 and 101–104 for which peak doubling was observed in the 2D [^15^N,^1^H]-HSQC spectrum of Fig. 7 ▶(*a*). The structure containing *trans* Pro104 is shown. C atoms are colored yellow (residues 64–66) or green (residues 101–104), N atoms are colored blue and O atoms are colored red. Stretches of polypeptide backbone outside of these segments are colored gray. (*b*) Surface view of the two segments in (*a*) colored in yellow and blue, respectively. (*c*, *d*) Identification of the *cis* and *trans* conformations in NP_247299.1 based on ^13^C chemical shifts and ^1^H–^1^H NOEs. (*c*) and (*d*) show strips from a 3D ^13^C(ali)-resolved [^1^H,^1^H]-NOESY spectrum representing the signals of the *trans* and *cis* forms of the Asn103–Pro104 peptide bond, respectively. (*c*) The *trans* form of Pro104 is manifested by *d*_αδ_^NP^ NOE connectivities (red arrows) and by the typical ^13^C^β^ and ^13^C^γ^ chemical shift pattern (blue arrows). (*d*) The *cis* form of Pro104 is manifested by *d*_αα_^NP^ NOE peaks (red arrows) and the large difference of about 9 p.p.m. between the ^13^C^β^ and ^13^C^γ^ chemical shifts (blue arrows).

*Cis*–*trans* isomerization of the C-terminal Asn–Pro peptide bond was first identified from analysis of the 2D [^15^N,^1^H]-HSQC spectrum (Fig. 7 ▶ *a*). Each isoform showed distinct peaks for Ile101, Ser102 and Asn103 and also for the spatially proximal Ile64, Ser65 and Glu66 (Figs. 8 ▶ *a* and 8 ▶ *b*). Interestingly, the residues Ile64–Glu66 also show higher 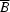-value-derived displacements in the crystal structure, which would appear to be a consequence of the influence of the Pro104 *cis*–*trans* equilibrium. The identity of the *cis* and *trans* isoforms was established from the characteristic ^13^C^β^ and ^13^C^γ^ chemical shifts (Grathwohl & Wüthrich, 1976*a* ▶) and from having either *d*_αδ_^NP^ or *d*_αα_^NP^ NOE connectivities, as illustrated in Figs. 8 ▶(*c*) and 8 ▶(*d*). In Fig. 8 ▶( ▶ *c*) strong NOE cross-peaks between H^δ2/3^ of Pro104 and H^α^ of Asn103 indicate that these are in close contact, as expected for a *trans* proline, while Fig. 8 ▶(*d*) shows the typical NOE pattern for a *cis* proline with strong peaks between H^α^ of Pro104 and H^α^ of Asn103 (Wüthrich, 1986 ▶). The amount of *cis* proline was estimated from the relative peak-intensity ratios of resonances belonging to the two isoforms to be about 25% (segments 101–104 and 66–68 both give equivalent values), which is in agreement with previous observations on C-­terminal proline *cis*–*trans* equilibria (Grathwohl & Wüthrich, 1976*b* ▶).

### 4.7. Further evaluation of the crystal structure in light of the NMR structure

In all of the aforementioned polypeptide segments for which the NMR data indicate local polymorphisms, there is no indication of multiple conformations based on the coordinates and structure factors deposited in the PDB. In order to more precisely define the range of ‘thermal motion/positional uncertainty’ in each of these regions that had higher *B* values than the rest of the structure, the X-­ray structure was subjected to multiple cycles of simulated-annealing refinement using *phenix.refine*. Except for residues in the 30–34 loop region (see below) and SeMet1, no significant differences relative to the original *REFMAC*5-refined coordinates were identified. In particular, for residues 10–15, the electron density is unambiguous and does not show any evidence of the Asp11–Val12 peptide flip indicated by the NMR data. Residues 30–34 did show some minor differences between the initial *REFMAC*5 model and the rebuilt *phenix.refine* model, but again there was no indication that the backbone adopts multiple conformations. For residues 36–39, where the NMR data are interpreted as a ‘sliding β-bulge’, the maps are once again unambiguous, without any indication of multiple conformations of the backbone. Multiple attempts were made at building the C-terminal tripeptide and the spatially adjoining segment of residues 64–66 into two conformations, representing the *trans* and *cis* isomers of Pro104 at varying occupancies, in order to investigate whether a small proportion of the *cis* isomer could be accommodated into the X-ray model. While there is clear-cut NMR evidence for the presence of two local conformations formed by these six residues (Figs. 7 ▶ and 8 ▶), we found no evidence for multiple conformations; the conformer with *cis*-Pro104 did not refine well and was pushed out of the density in each case. It should be noted that this region packs against a symmetry-related molecule in the crystal structure. Moreover, the carboxy-terminus hydrogen bonds to Lys36 in the symmetry-related molecule and to two waters, which together appear to stabilize the *trans* conformation.

Overall, there is no evidence for local multiple conformations in the X-ray electron-density maps recorded at 1.7 Å resolution and 100 K that would correspond to the NMR observations in solution at 313 K. This leads to the conclusion that one predominant conformation is present at 100 K and in the crystal lattice, but that the locally increased *B* values in these few specific regions are indicative of some static or dynamic polymorphism that can be observed in more detail in the solution NMR measurements at a much higher temperature. A recent related assessment of dynamics in the crystal at low temp­erature and correlations with solution NMR data concluded that additional ambient-temperature X-ray data collection combined with mutagenesis could aid in uncovering relations to function of subtle correlations between multiple data sets recorded with different methods (Fraser *et al.*, 2009 ▶).

## Supplementary Material

PDB reference: NP_247299.1, 2kla

## Figures and Tables

**Table 1:** Determination of the NMR structure, a reference crystal structure and a reference NMR structure of the protein NP_247299.1: input for the structure calculations and characterization of bundles of 20 energy-minimized *CYANA* conformers representing the structures

	Value†		
Quantity	NMR structure‡	Reference crystal structure§	Reference NMR structure¶
NOE upper distance limits	2452	4523	4209
Intra-residual	569	1021	1194
Short-range	749	1073	1033
Medium-range	130	819	726
Long-range	1004	1610	1256
Dihedral angle constraints	432	392	409
Residual target-function value (Å^2^)	1.80 ± 0.25	1.09 ± 0.18	1.08 ± 0.11
Residual NOE violations			
No. ≥0.1 Å	21 ± 5	4 ± 2	1 ± 1
Maximum (Å)	0.18 ± 0.10	0.12 ± 0.05	0.11 ± 0.02
Residual dihedral angle violations			
No. ≥2.5°	1 ± 1	0 ± 1	0 ± 1
Maximum (°)	3.67 ± 2.60	1.07 ± 0.91	1.97 ± 0.66
AMBER energies (kcal mol^−1^††)			
Total	−4124 ± 121	−4293 ± 84	−4227 ± 97
van der Waals	−362 ± 15	−403 ± 10	−381 ± 9
Electrostatic	−4598 ± 107	−4643 ± 79	−4631 ± 90
R.m.s.d. from mean coordinates‡‡ (Å)			
Backbone (1–102)	0.48 ± 0.06	0.32 ± 0.05	0.48 ± 0.07
All heavy atoms (1–102)	0.89 ± 0.08	0.63 ± 0.05	0.88 ± 0.08
Ramachandran plot statistics§§			
Most favored regions (%)	79.9	87.6	80.0
Additional allowed regions (%)	17.6	12.3	17.6
Generously allowed regions (%)	1.8	0.1	1.7
Disallowed regions (%)	0.7	0.0	0.7

† Except for the top six entries, average values and standard deviations for the 20 energy-minimized conformers are given.

‡ Structure calculated from the experimental NMR data. The top six entries represent the input generated in the final cycle of the *UNIO-ATNOS*/*CANDID* and *CYANA* calculations (see text for details).

§ Structure calculated with *CYANA* from conformational constraints derived from the molecular model representing the crystal structure (see text for details).

¶ Structure calculated with *CYANA* from conformational constraints derived from the bundle of 20 molecular models representing the NMR structure (see text for details).

†† 1 kcal mol^−1^ = 4.186 kJ mol^−1^.

‡‡ The numbers in parentheses indicate the residues for which the r.m.s.d. was calculated.

§§ As determined by *PROCHECK* (Laskowski *et al.*, 1993 ▶). The crystal structure (2qtd) deposited in the PDB has values of 92.9% favored, 7.1% additionally allowed, 0% generously allowed and 0% disallowed.

**Table 2:** Backbone ϕ dihedral angle values for residues 10–15 and 30–35 of NP_247299.1 See text for the residue selection.

	ϕ (°)/^3^*J*_HNα_ (Hz)		
Residue	NMR structure†	Crystal structure‡	^3^*J*_HNα_ measured[Table-fn tfn10] (Hz)
Met10	−116/10.0	−118/10.1	8.9
Asp11	−101/9.3	−96/8.8	9.0
Val12	−143/8.9	−63/4.5	7.1
Asp13	−127/10.0	−108/9.7	n.d.[Table-fn tfn11]
Lys14	−152/7.9	−175/4.8	n.d.[Table-fn tfn11]
Ile15	−58/3.9	−60/4.2	4.8
Ile30	−110/9.8	−113/10.0	10.7
Asp31	−137/9.4	−145/8.7	7.8
Asp32	−165/6.1	40/6.6	7.8
Asn33	59/7.3	56/7.3	6.5
Glu34	−124/10.0	−130/9.8	8.0
Val35	−70/5.4	−66/4.9	4.7

† ϕ-angle values in the NMR structure expressed as the average from the 20 conformers and corresponding values for ^3^ *J*_HNα_ predicted by the Karplus relation.

‡ ϕ-angle values in the crystal structure and corresponding values for ^3^ *J*_HNα_ predicted by the Karplus relation.

§ Experimentally measured ^3^ *J*_HNα_ coupling constants.

¶ Precise values could not be determined owing to spectral overlap.
